# A 3T Magnetic Resonance Fractional Order Calculus Model for the Diagnosis and Grading of Endometrial Cancer

**DOI:** 10.2174/0115734056419307260514062806

**Published:** 2026-05-24

**Authors:** Shu-Yuan Li, Qing-Lei Shi, Yu-Xuan Shao, Ya Su, Shi-Feng Xiang, Yi-Ping Wu, Bulang Gao

**Affiliations:** 1 Department of Radiology, Anhua County People’s Hospital, Anhua, China; 2 Chinese University of Hong Kong (Shenzhen) School of Medicine and Shenzhen Research Institute of Big Data, Shenzhen, China; 3 Hebei North University, Zhangjiakou, China; 4 Department of CT / MRI, Handan Central Hospital, Handan, China

**Keywords:** Magnetic resonance imaging, Endometrial cancer, Fractional order calculus model, Radiomics, Pathological grades, high-calorie diet

## Abstract

**Introduction:**

To investigate MRI fractional order calculus model (FROC) on distinguishing different pathological grades of endometrial carcinoma (EC).

**Methods:**

Fifty patients with pathologically-confirmed EC and normal volunteers were enrolled for analysis of parameters β, μ, and D.

**Results:**

The parameters β, μ, and D of the EC group were all significantly (*P*<0.05) lower than the normal controls. Parameters β, μ, and D had high diagnostic efficacy in distinguishing EC, with the AUC of β, μ, and D being 0.777, 0.660, and 0.809, respectively. The β Energy and Total Energy were significantly (*P*<0.05) different between G1 and G3 pathological grades of EC. The β Energy, Kurtosis, and Total Energy were significantly (*P*<0.05) different between G2 and G3 pathological grades and had AUCs of 0.730, 0.795, and 0.812, respectively. The D Entropy, Total Energy, and Energy showed a significant (*P*<0.05) difference between G2 and G3 grades and had AUCs of 0.724, 0.761, and 0.756, respectively. The μ Energy, Entropy, and Total Energy showed a significant (*P*<0.05) difference between G2 and G3 grades.

**Discussion:**

The use of parameters of the FROC model is effective for the diagnosis of endometrial carcinoma. Nonetheless, the limitations should be properly addressed, with validation of the model in a larger, multi-center cohort, incorporating second-order and high-order texture features.

**Conclusion:**

The use of parameters of the FROC model is effective for the diagnosis of EC, and the first-order statistical parameters have high clinical value in differentiating high- and low-grade EC.

## 1 INTRODUCTION

Endometrial carcinoma is one of the most common gynecological malignant tumors in developed countries worldwide [1, 2]. Based on data from the National Cancer Center in 2022, the incidence of endometrial carcinoma in China was 10.54/100,000 and the mortality rate was 2.53/100,000 [1]. With the increase in living standards and changes in lifestyle toward a high-fat, high-sugar, and high-calorie diet and low physical activity, the incidence of endometrial carcinoma has significantly increased in China [3]. Early diagnosis and treatment are key to successful treatment and better prognosis.

Magnetic resonance imaging (MRI) has been widely applied for preoperative examination of endometrial cancer due to its high soft tissue resolution. Diffusion weighted imaging (DWI) is an imaging method for assessing the diffusion and restriction of water molecules in tissues and lesions, which can reflect the pathological and physiological characteristics of the internal tissue structures [4]. However, DWI is often obtained under a single b-value and cannot further reflect the material basis that causes velocity changes, such as diffusion spatial distance. The MRI fractional order calculus (FROC) model covers 11 b-values from low to high b-values and can more accurately and comprehensively reflect the internal conditions of the tissue. It has been used for evaluation of children's brain tumors, bladder cancer, breast cancer, cervical carcinoma, and renal cell carcinoma [5-9]. Imaging omics can quantify image information into meaningful data, quantitatively reflect the pathophysiology within tissues, and is widely used in oncological lesions and other fields [10]. However, studies using the MRI FROC model for assessment of endometrial carcinoma are currently unavailable. The aim of this study was to explore the value of the β, μ, and D first-order statistical parameters of the FROC model and related imaging omics for the diagnosis and grading of endometrial carcinoma.

## 2 MATERIALS AND METHODS

This retrospective single-center study was approved by the ethics committee of Handan Central Hospital (Handan, China, 20220288), and informed consent was waived by the same ethics committee because of the retrospective study design. The Helsinki Declaration has been followed in this study. All participants had the opportunity to opt out per the Helsinki Declaration. All methods were performed in accordance with the relevant guidelines and regulations, including the STROBE and STARD guidelines. Patients (women) with endometrial cancer who underwent MRI examination from March 2022 to December 2023 were enrolled.

The inclusion criteria were patients with endometrial cancer who underwent MRI scans with multiple b-value sequences for preoperative diagnosis and had postoperative pathological confirmation of endometrial cancer. According to the 2023 Federation of Gynecology and Obstetrics (FIGO) pathological grading criteria for endometrial carcinoma [11], the patients were divided into G1 (low-grade group), G2 (low-grade group), and G3 (high-grade group). Meanwhile, volunteers with normal endometrium were enrolled as the control group.

The exclusion criteria were patients with incomplete MRI data, lack of multiple b-value sequences, lack of postoperative pathological results, preoperative radiotherapy, chemotherapy or other treatments, poor image resolution, and significant artifacts that prevented proper diagnosis.

### 2.1 Equipment and Scanning Method

All subjects were scanned using the same Siemens Magnetom Skyra 3.0T magnetic resonance scanner and an 18 channel-phased array system (Siemens, Germany), with a scanning range from the sacroiliac joint to the pubic symphysis level. The scanning parameters were: (1) cross-sectional T2WI: fs-FRF SE, TR/TE 3896 ms/71.7ms, 5thk/6sp, field of view (FOV) 300 × 225, matrix 256 × 256; (2) Sagittal plane T2WI: fs-FSE, TR/TE 3516 ms/69 ms, 5thk/6sp, FOV215 × 270, matrix 256 × 256; (3) Cross-section T1WI: FSE, TR/TE637 ms/9.1ms, 5thk/6sp, FOV280 × 210, matrix 256 × 256; (4) Coronal T2WI: WATER IDEAL, TR/TE1997ms/68.6ms, 5thk/6sp, FOV 280 × 210, matrix 256 × 256; (5) Multiple b-value DWI scanning: reduced FOV, Freq FOV: 28, Phase FOV: 0.35-0.5, reduced matrix, changed frequency encoding direction, and 11 b-values at one scan, with b-values of 0, 50, 100, 150, 200, 300, 400, 800, 1200, 1600, and 2000s/mm^2^.

### 2.2 Imaging Process

The imaging was evaluated without knowledge of the clinical conditions by two senior radiologists experienced in the diagnosis of gynecological diseases (with 5 and 10 years of imaging diagnosis and treatment experience, respectively).

Firstly, the region of interest (ROI) was drawn in a double-blind experimental setting. Two experienced imaging experts used the ITK-SNAP tool to manually delineate the ROI layer by layer on all imaging data. Using axial T2WI as a reference, the range of tumor solid components was defined on high b-value DWI images. The ROI included all tumor solid components as much as possible, avoiding areas such as necrosis and cystic changes. After completing the delineation of the ROI for all sequential images, radiological experts conducted a review and made necessary corrections to ensure the formation of accurate 3D voxel images of the tumor.

Secondly, parameter value processing was performed using MATLAB R2021a (MathWorks, Natick, Massachusetts, USA) to process the DWI and FROC data and fit the FROC model using the following formula:


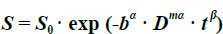


This generated pseudo-color maps of the diffusion coefficient (D), voxel diffusion heterogeneity parameter (β), and spatial constant (μ), respectively.

### 2.3 Extraction of Image Omics Features

The radiomics feature values from images of different pathological grades of endometrial carcinoma were extracted using the open-source third-party software package Pyradiomics. Due to the small amount of case data, the extracted feature values mainly consisted of first-order statistics, including Median, Minimum, Range, Robust Mean Absolute Deviation, Root Mean Squared, 10th Percentile, 90th Percentile, Energy, Entropy, Interquartile Range, Kurtosis, Maximum, Mean Absolute Deviation, Uniformity, Variance, Mean, Skewness, and Total Energy, totaling 18 feature values.

### 2.4 Statistical Analysis

The data were statistically analyzed using SPSS 25.0 software (IBM, Chicago, IL, USA). For a two-tailed test, with an effect size of 0.5, an alpha error probability of 0.05, and a power (1–β error probability) of 0.95, at least 42 patients were required for this study. The Shapiro–Wilk test was used to evaluate whether the quantitative data conformed to a normal distribution, and Levene’s test was used to test for homogeneity of variance. The LSD test was used for inter-group comparison. Data that conformed to a normal distribution were represented as mean ± standard deviation, while data that did not conform to a normal distribution were represented as median and interquartile range (P25, P75). The quantitative data of various parameters between patients and normal volunteers, as well as the inter-group comparison of first-order statistics at different pathological grades, were analyzed using the Mann–Whitney U test or independent-samples t-test. The differences in various parameters at different pathological grades were analyzed using the Kruskal–Wallis H test or one-way ANOVA. Receiver operating characteristic (ROC) curve analysis was performed for parameters with statistical significance, with calculation of diagnostic performance, including the area under the ROC curve (AUC). Consistency analysis of FROC parameters measured blindly in the ROI by two physicians was evaluated using the intra-class correlation coefficient (ICC), with ICC ≥ 0.75 indicating good consistency. A *p*-value < 0.05 was considered statistically significant.

## 3 RESULTS

### 3.1 Subjects

Fifty women with endometrial carcinoma aged 56.0±7.9 years were enrolled and divided into group G1 (low-grade group, n=5), G2 (low-grade group, n=33), and G3 (high-grade group, n=12). Meanwhile, 30 volunteers aged 55.4 ± 9.0 years with normal endometrium were enrolled as the control group.

### 3.2 Statistical Analysis of the FROC Model Parameters

**Fig. (1):**
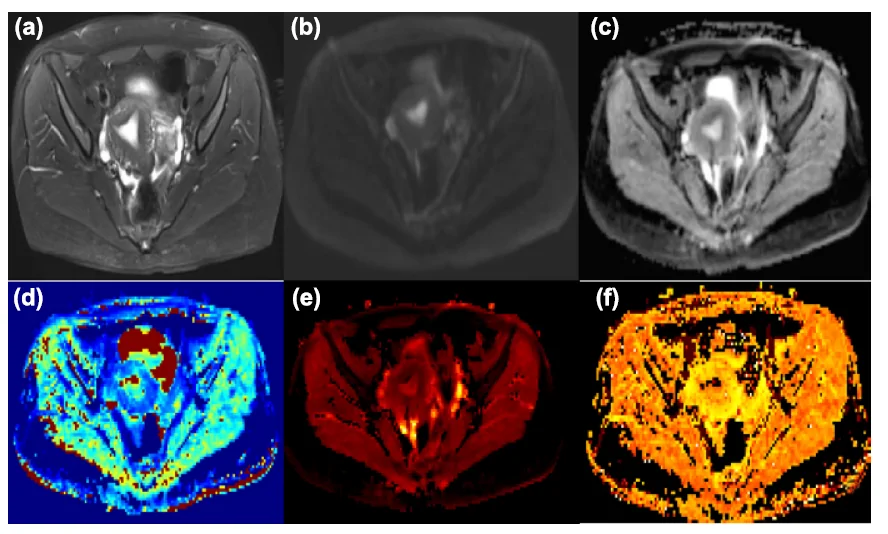
Normal endometrium with pseudo-color images of different fractional order calculus model (FROC) model parameters in a female patient aged 51 years. A-C. T2WI (**a**), DWI (**b**), and apparent diffusion coefficient (**c**) images were present. (**d**-**f**). The pseudo-color images of FROC model parameters β (**d**), parameter D (**e**), and parameter μ (**f**) were present.

**Fig. (2):**
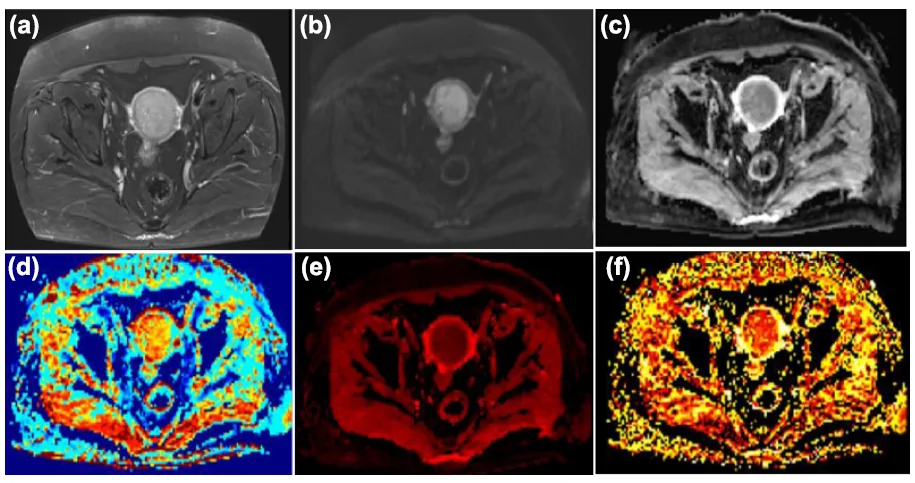
A 68-year-old woman was diagnosed with endometrial carcinoma with the pathological grade of G3. A-C. Transverse T2 weighted imaging (T2WI) in figure (**a**), diffusion weighted imaging (DWI) in figure (**b**), and apparent diffusion coefficient (ADC) images in figure (**c**). D-F. Pseudo color images for the parameters of the fractional order calculus (FROC) model β (**d**), D (**e**), and μ (**f**).

The parameters β, μ, and D of the endometrial carcinoma group were all significantly (*P* < 0.05) lower than those of the normal controls (Figs. **1**, **2** and Table **1**). ROC curve analysis revealed that parameters β, μ, and D had high diagnostic efficacy in distinguishing between endometrial carcinoma and normal endometrium, with AUC values of 0.777, 0.660, and 0.809, respectively. The parameter D had the maximum AUC of 0.809 (0.702–0.891). The Wilcoxon rank test was used for inter-group comparison of parameters in FROC models with different pathological grades of EC (endometrial carcinoma), and there was no statistically significant difference in the parameters β, D, and μ of the FROC model between different EC groups (Table **2**). The inter-observer agreement of data measurement (β, D, and μ) was high, with an ICC of 0.89 (> 0.75), and the mean value was used for final analysis.

### 3.3 First-order Statistical Parameters

**Fig. (3):**
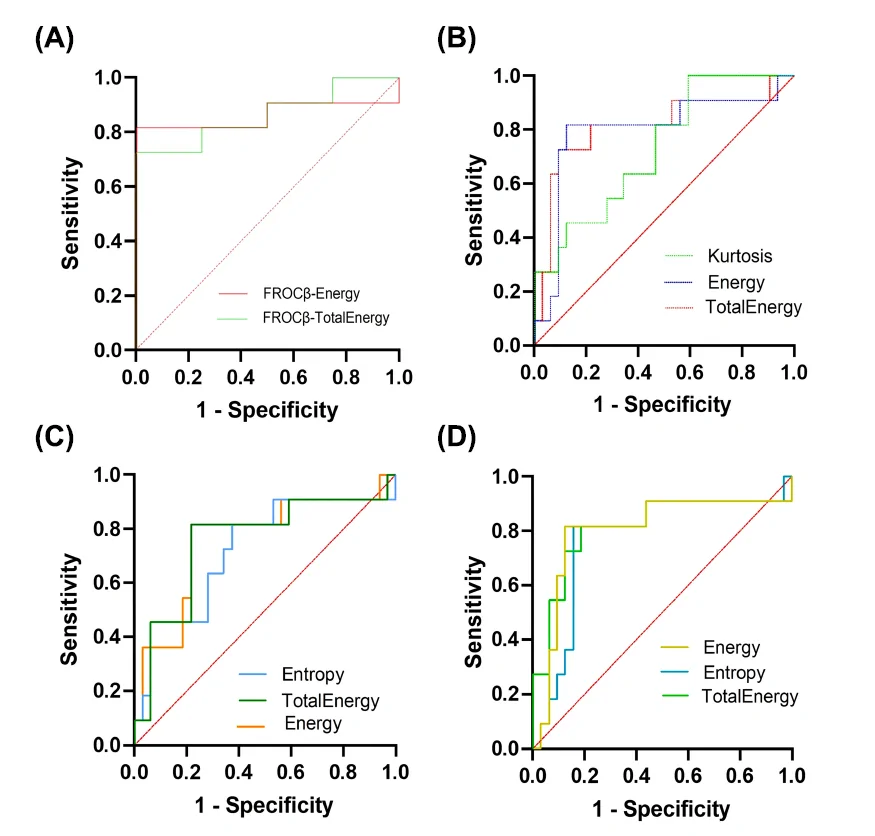
Receiver operator characteristics (ROC) curve analysis of first-order statistical parameters (β, D, and μ) of the fractional order calculus (FROC) models at different pathological grades of endometrial carcinoma (EC). **a**. The Energy and Total Energy of the β first-order statistics, which had a significant (*P*<0.05) difference between G1 and G3 pathological grades of EC, had the same area under the ROC curve (AUC) of 0.864. **b**. The Kurtosis, Energy, and Total Energy of the β first-order statistics, which had a significant (*P*<0.05) difference between G2 and G3 grades, had an AUC of 0.730, 0.795, and 0.812, respectively. **c**. The Entropy, Total Energy, and Energy of the D first-order statistics, which had a significant (*P*<0.05) difference between G2 and G3 grades, had an AUC of 0.724, 0.761, and 0.756, respectively. **d**. The Energy, Entropy, and Total Energy of the μ first-order statistics level, which had a significant (*P*<0.05) difference between G2 and G3 grades, had an AUC of 0.801, 0.773, and 0.815, respectively.

No statistically significant (*P*>0.05) difference was detected in any first-order statistical parameter between G1 and G2 pathological grades of endometrial carcinoma. The Energy and Total Energy of the β first-order statistical parameter were significantly (*P*<0.05) different between G1 and G3 pathological grades of endometrial carcinoma, both with an AUC of 0.864 (Fig. **3a**). The Energy, Kurtosis, and Total Energy of the β first-order statistical parameter were significantly (*P*<0.05) different between G2 and G3 pathological grades (Tables **3** and **4** and Fig. **3b**) and had an AUC of 0.730, 0.795, and 0.812, respectively. The Entropy, Total Energy, and Energy of the D first-order statistical parameter had a significant (*P*<0.05) difference between G2 and G3 grades and had an AUC of 0.724, 0.761, and 0.756, respectively (Tables **3** and **4** and Fig. **3c**). The Energy, Entropy, and Total Energy of the μ first-order statistical parameter had a significant (*P*<0.05) difference between G2 and G3 grades and had an AUC of 0.801, 0.773, and 0.815, respectively (Tables **3**-**5** and Fig. **3d**).

## 4 DISCUSSION

After investigating the effect of the MRI FORC model on distinguishing endometrial carcinoma from normal endometrium and on distinguishing different pathological grades of endometrial carcinoma, it was found that the use of the parameters of the FROC model was effective for the diagnosis of EC, and the first-order statistical parameters of the radiomics had high clinical values in differentiating high- and low-grade endometrial carcinoma.

The DWI uses changes in the movement of water molecules within tissues to reflect the density of cells. Apparent diffusion coefficient (ADC), as a quantitative parameter of DWI, has been widely used in clinical practice. The conventional single ADC index model is based on the assumption that human tissues are homogeneous substances; however, the actual human body is composed of complex tissue components. Thus, the conventional single index model cannot reflect changes in more tissue structure information. In addition, ADC is often obtained at a single low b-value, which cannot reflect the speed of water molecule diffusion and thus cannot reflect the material conditions that cause speed changes. Therefore, in order to obtain more accurate water diffusion information and map the microstructure of tissues, non-Gaussian diffusion models were derived based on the DWI data, including FROC, voxel incoherent motion (IVIM), continuous time random walk (CTRW), diffusion kurtosis imaging (DKI), and stretched exponential model (SEM). Each non-Gaussian diffusion model has its own advantages and characteristics, and FROC has a greater advantage in distinguishing between low-grade and high-grade tumors [10-14]. Nonetheless, it is a direct indicator for evaluating tumor biological behavior. The FROC model mainly includes three parameters [12-14]: β, D, and μ. Parameter β represents the uniformity within the organization, with a range of (0,1). The closer the parameter β is to 0, the more complex the internal components of the organization are. If β is equal to 1, it indicates that the internal composition of the organization is uniform; in this case, the FROC model can be considered as a single exponential model. The parameter D represents the diffusion rate of water molecules. The parameter μ is a spatial parameter that is related to the distance of free diffusion of water molecules. Studies have shown that as the b-value increases, the changes in tissue signals do not decrease according to a single exponential law [15, 16]. In addition, the movement of water molecules does not follow the Gaussian distribution [17], which further confirms the limitations of ADCs. The FROC model is based on the stretching index model and provides a detailed description of human tissues. In this study, the FROC model covers 11 b-values from low to high, and can quantitatively describe the changes in tissue and lesion signals, thereby reflecting the biological behavior of malignant tumors. At present, it has been widely used for tumor diseases, including children's brain tumors, bladder cancer, kidney cancer, and others [6, 7, 12].

Our study used a high b-value diffusion model, with a b-value range of 0-2000s/mm^2^. The parameters of the FROC model were used to distinguish between endometrial carcinoma and normal endometrium, and the normal endometrium group had significantly higher β, D, and μ values than those of the endometrial carcinoma group, similar to the results of other studies [7, 12]. In our study, the endometrial carcinoma β value is significantly lower than that of the normal endometrial group, and the internal tissue components of endometrial carcinoma are complex and prone to cystic changes and necrosis, leading to a low β value, which is similar to the outcome of the study by Luo *et al*. [18]. The normal endometrial group had significantly higher D and μ values than those in the endometrial carcinoma group, which may be related to the fast growth rate of the endometrial carcinoma cells, high cell density, slow diffusion rate of water molecules [18], and short distances. Our study also used the D and μ values to distinguish different pathological grades of endometrial carcinoma, and the results showed no significant statistical difference among the parameters of the FROC model in distinguishing different pathological grades, which may be related to factors such as small sample size and internal heterogeneity of the lesion [18]. Scholars Shao *et al*. [5] and Zhang *et al*. [19] have concluded that the only parameter β in the FROC model can be used to distinguish cervical adenocarcinoma and cervical squamous cell carcinoma, but the studies by Shao *et al*. [5] also showed that various parameters could be used to distinguish between high and low grades of cervical cancer.

Brain MRI imaging contains complex data that pose great challenges for computational analysis, and although models proposed for brain MRI analysis produce encouraging results, the high complexity of neuroimaging data may hinder generalizability and clinical application [20, 21]. Moreover, MRI, as an imaging examination method, is susceptible to subjective interpretation, with significant differences among observers and a lack of objective and quantitative evaluation criteria. The concept of radiomics was officially proposed by Lambin *et al*. in 2012 [22], which involves automated and high-throughput feature extraction to transform images into usable data features. Researchers [23, 24] have enhanced the U-Net with attention mechanisms for improved feature representation in lung nodule segmentation. Imaging omics is a novel method that utilizes image analysis to obtain reproducible data through high-throughput and automatic quantification of standard medical images, including complex information that is difficult to observe or quantify by the human eye, thereby transforming image information into data [25, 26]. Imaging omics is widely used in endometrial carcinoma, including the assessment of myometrial invasion.

The combination of MRI imaging omics and imaging features has high diagnostic value for histological grading of endometrial carcinoma [25, 26]. The imaging omics nomogram based on MRI multiparametric data has higher value in predicting preoperative endometrial carcinoma grading compared with curettage [27]. Zheng *et al*. showed that the combined imaging omics model has greater advantages in predicting high-grade endometrial carcinoma [28]. In addition, texture analysis helps predict high-grade endometrial carcinoma [29].

Our study conducted statistical analysis on first-order variables based on the FROC model and found significant differences in two to three first-order statistical parameters between grades 1 and 3 or between grades 2 and 3 of endometrial carcinoma. First-order statistics in radiomics describe the distribution of voxel intensity within the ROI in an image without considering the spatial relationships between voxels. This study analyzed first-order features of the ROI and selected four optimal parameters. The four features—Energy, Total Energy, Entropy, and Kurtosis—had significant clinical and biological relevance because they quantify tumor heterogeneity and cellular activity within endometrial carcinoma from different perspectives, which are closely related to tumor invasiveness, grading, prognosis, and treatment response. Zheng *et al*. found that preoperative radiomics analysis based on MRI can predict high-grade EC [28].

The analysis of first-order radiomics features of three-dimensional whole tumor volume is of great significance. On the one hand, this is because radiomics features from three-dimensional whole tumor volume MRI have good repeatability, and on the other hand, the extraction of first-order features from the whole tumor can better reflect tumor heterogeneity.

The sample size in our current study was relatively small, with a total of 50 participants included, which may affect the stability and generalizability of the model. This is because the disease is rare, and a larger cohort of such patients cannot easily be enrolled, which reduces the statistical reliability of comparisons involving the G1 group (such as G1 *vs*. G3). We fully recognize that this presents certain limitations in statistical power, and this study should therefore be treated as exploratory and preliminary in nature.

Moreover, the sample size is uneven among the groups, especially with only 5 patients in the G1 group due to the rarity and difficulty of pathological diagnosis of this disease, which makes it difficult to recruit such cases. The significant result (AUC = 0.864) obtained from this comparison should be treated as a preliminary finding, interpreted cautiously, and validated in a larger, balanced cohort. In the future, these issues will be addressed through large-sample, multicenter, balanced studies.

Although the number of enrolled G1 cases was small, the impact on the combined variance estimation was limited, and the mean estimation error remained within a controllable range. This study only provided preliminary research data and did not include multimodal MRI data. In the future, a multimodal MRI diagnostic model should be established to further improve model performance.

The delineation of lesions is currently mainly performed manually, which is time-consuming and has a certain degree of subjectivity. Subsequently, semi-automatic or automated methods will be introduced to improve the accuracy and repeatability of delineation. Finally, the model should be validated in a larger multicenter cohort, incorporating second-order texture features, and machine learning classifiers should be used to construct a prediction model for repeated validation with larger samples.

This study was designed as a single-center exploratory study aiming at proposing hypotheses and identifying potential differential signals between different grades of tumors. Given the strict inclusion criteria and the rarity of G1 group cases of endometrial cancer studied, recruiting a large sample in the early stages was challenging. We have conducted efficacy analysis based on the observed effect size in the main comparison. For the main comparison between the entire patient population (n=50) and the control group (n=30), our analysis showed that the effect size was achieved (Table **2**).

Point-of-care photography in clinics using mobile devices is a new standard of care for clinical documentation, and high-quality cameras in modern smartphones may facilitate faithful reproduction of clinical findings in photographs. Nevertheless, clinical photographs captured on mobile devices are often taken using the native camera application on the device and transmitted using relatively insecure methods [30]. Thus, an internally developed mobile application may be launched to allow healthcare providers to securely capture clinical photographs and upload them to the intranet in a manner compliant with patient privacy and confidentiality regulations.

In this study, we used only the native intranet system to obtain the relevant MRI images without using any imaging data that could compromise patient privacy; therefore, patient privacy was well preserved.

## 5 LIMITATIONS

Some limitations existed in this study, including the retrospective, single-center design; a small cohort of patients, especially for subgroup analysis; inclusion of Chinese patients only; limited analytical methods using mainly univariate analysis and first-order features for AUC value reporting; no randomization; no external validation; and non-inclusion of second-order and higher-order texture features such as gray-level co-occurrence matrix (GLCM), gray-level run-length matrix (GLRLM), and gray-level size zone matrix (GLSZM), which may all affect the generalizability of the study outcomes.

The inclusion of radiomics feature sets such as GLCM, GLRLM, and GLSZM may more comprehensively quantify tumor heterogeneity and further improve the predictive performance and biological interpretability of the model. Future prospective, randomized, controlled, multicenter studies involving a large sample of patients from multiple races and ethnicities, with external validation and inclusion of second- and higher-order texture features, should be performed to develop a more robust multivariate joint prediction model.

## CONCLUSION

To sum up, the use of parameters of the FROC model is effective for the diagnosis of endometrial carcinoma, and the first-order statistical parameters of FROC radiomics have high clinical value in differentiating high- and low-grade endometrial carcinoma. Nonetheless, the limitations specific to this study should be properly addressed, with validation of the model in a larger, multicenter cohort, incorporating second-order and higher-order texture features, and using machine learning classifiers to build a multivariate joint prediction model rather than relying solely on univariate AUC reporting.

## Figures and Tables

**Table 1:** First-order parameters in the FROC model between controls and EC patients.

Variables	β	D (μm^2^ /ms)	μ (μm)
Normal control	0.841(0.785,0.892)	1.033(0.823,1.267)	3.388(2.870,3.644)
EC group	0.767(0.739,0.791)	0.756(0.668,0.855)	3.593(3.403,3.694)
Z	-3.987	-4.458	2.311
*P*	<0.001	<0.001	0.021

**Note:** FROC, fractional order calculus; EC, endometrial carcinoma.

**Table 2:** First-order parameters of the FROC model at different pathological grades of EC.

Grades	β	D (μm^2^ /ms)	μ (μm)
G1	0.764(0.661,0.775)	0.759(0.670,0.828)	3.625(3.602,3.731)
G2	0.769(0.739,0.811)	0.762(0.670,0.922)	3.568(3.395,3.690)
G3	0.755(0.730,0.788)	0.709(0.656,0.868)	3.608(3.281,3.788)
H	0.967	0.963	1.398
*P*	0.617	0.618	0.497

**Note:** FROC, fractional order calculus; EC, endometrial carcinoma.

**Table 3:** First-order statistical parameters of the FROC models at different pathological grades of EC.

Variables	G1 *vs*. G3			G2 *vs*. G3		
	***P***	**AUC**	**95%CI**	***P***	**AUC**	**95%CI**
β	-	-	-	-	-	-
Energy	0.040	0.864	0.671-1.000	0.003	0.795	0.616-0.975
Kurtosis	-	-	-	0.023	0.730	0.565-0.895
Total Energy	0.040	0.864	0.676-1.000	0.002	0.812	0.640-0.985
μ	-	-	-	-	-	-
Energy	-	-	-	0.002	0.801	0.621-0.982
Entropy	-	-	-	0.007	0.773	0.596-0.950
Total Energy	-	-	-	0.001	0.815	0.641-0.990
D	-	-	-	-	-	-
Energy	-	-	-	0.011	0.756	0.572-0.939
Entropy	-	-	-	0.027	0.724	0.537-0.912
Total Energy	-	-	-	0.009	0.761	0.583-0.940

**Note:** FROC, fractional order calculus; EC, endometrial carcinoma; AUC, area under the receiver operating characteristic curve; CI, confidence interval.

**Table 4:** Significant differences at the 2nd and 3rd levels in the Wilcoxon test of the first-order statistics.

Variables		Energy	Kurtosis	Total Energy
β	G2	140696077(76350912,286571037)	2.90(2.27,3.30)	5727737297(2775065131,12895704886)
	G3	509505779(393918060,685058182)	3.27(2.96,4.25)	22774167214(14286366200,2927941399)
	Z	-2.895	-2.255	-3.062
	*P*	0.004	0.024	0.002
*D*	G2	154078362(87038976,348418154)	6.17(5.63,6.64)	6847052181(3057889081,12565032428)
	G3	408291235(294743455,684941868)	6.47(6.35,6.97)	18135860005(13263449853,29205578233)
	Z	-2.505	-2.199	-2.561
	*P*	0.012	0.028	0.010
μ	G3	944937991(7244685577,13751752945)	7.50(7.43,7.87)	403663848712(273096061275,580310980022)
	Z	-2.951	-2.672	-3.090
	*P*	0.003	0.008	0.002

**Table 5:** Statistically significant first-order statistics of FROC, CTRW model parameters, and ADC in different pathological parameters.

Variables	G1 *vs*. G3			G2 *vs*. G3		
	**AUC**	**95% CI**	**P**	**AUC**	**95% CI**	**P**
FROCβ	-	-	-	-	-	-
Energy	0.864	0.671-1.000	0.040	0.795	0.616-0.975	0.003
Kurtosis	-	-	-	0.730	0.565-0.895	0.023
Total Energy	0.864	0.676-1.000	0.040	0.812	0.640-0.985	0.002
FROCμ	-	-	-	-	-	-
Energy	-	-	-	0.801	0.621-0.982	0.002
Entropy	-	-	-	0.773	0.596-0.950	0.007
Total Energy	-	-	-	0.815	0.641-0.990	0.001
FROCD	-	-	-	-	-	-
Energy	-	-	-	0.756	0.572-0.939	0.011
Entropy	-	-	-	0.724	0.537-0.912	0.027
Total Energy	-	-	-	0.761	0.583-0.940	0.009
CTRWα	-	-	-	-	-	-
Entropy	0.886	0.670-1.000	0.007	0.707	0.549-0.866	0.046
Energy	-	-	-	0.793	0.611-0.974	0.003
Total Energy	-	-	-	0.801	0.625-0.977	0.002
CTRWβ	-	-	-	-	-	-
Energy	0.864	0.671-1.000	0.040	0.793	0.611-0.974	0.003
Total Energy	0.864	0.676-1.000	0.040	0.812	0.642-0.983	0.002
Kurtosis	-	-	-	0.804	0.665-0.943	0.002
CTRWDm	-	-	-	-	-	-
Energy	-	-	-	0.753	0.569-0.937	0.012
Entropy	-	-	-	0.753	0.596-0.937	0.012
Total Energy	-	-	-	0.761	0.583-0.940	0.009
ADC	-	-	-	-	-	-
Energy	-	-	-	0.759	0.575-0.942	0.010
Entropy	-	-	-	0.736	0.550-0.922	0.020
Total Energy	-	-	-	0.767	0.589-0.945	0.008

**Note:** FROC, fractional order calculus; EC, endometrial carcinoma; AUC, area under the receiver operating characteristic curve; CI, confidence interval; ADC, apparent diffusion coefficient.

## Data Availability

The data of current study are available from corresponding author, [S-F.X] and [Y-P.W] on a reasonable request.

## LIST OF ABBREVIATIONS

MRI: Magnetic Resonance Imaging
FROC: Fractional Order Calculus Model
EC: Endometrial Carcinoma
ROC: Receiver Operating Characteristic(s)
AUC: Area Under the ROC Curve
DWI: Diffusion-Weighted Imaging
FIGO: Federation of Gynecology and Obstetrics
FOV: Field of View
ADC: Apparent Diffusion Coefficient
IVIM: Intravoxel Incoherent Motion
CTRW: Continuous Time Random Walk
DKI: Diffusion Kurtosis Imaging
SEM: Stretched Exponential Model
GLCM: Gray-Level Co-occurrence Matrix
GLRLM: Gray Level Run Length Matrix
GLSZM: Gray Level Size Zone Matrix

## STANDARDS OF REPORTING

STROBE and STARD guidelines were followed.
